# Supplementary material for: Spatial Variability of COVID-19 Hospitalization in the Silesian Region, Poland
Source: Int J Environ Res Public Health. 2022 Jul 25;19(15):9007. doi: 10.3390/ijerph19159007 (PMC9331287; doi:10.3390/ijerph19159007)
Supplement: Supplementary file 1 [file ijerph-19-09007-s001.zip › ijerph-1809926-supplementary.pdf]

International Journal of
Environmental Research and Public Health

MDPI

Supplement 1

A. Number of hospitalized due to COVID-19 and dead COVID-19 patients, values of crude and standardized rates of morbidity due to COVID-19 (n/100,000), and values of in-hospital mortality due to COVID-19 in the Silesian Voivodeship in 2020 by poviats

Year		2020										
Code of poviat	Poviat	Number of hospitalized patients	Crude morbidity rate [n/100,000 inhabitants]	Standardized morbidity rate [n/100,000 inhabitants]	Number of dead patients	In-hospital mortality [%]	Number of hospitalized patients age <65 years	Number of hospitalized patients age 65+ years	Number of dead patients without comorbidities	Number of dead patients with one coexisting disease	Number of dead patients with two coexisting disease	Number of dead patients with three coexisting disease
2401	będziński	404	274.3	188.8	92	22.8%	180	224	33	33	9	17
2402	bielski	523	314.4	240.5	87	16.6%	211	312	20	18	17	32
2461	Bielsko-Biała	555	326.9	211.0	102	18.4%	175	380	19	14	30	39
2414	bieruński-lędziński	125	208.9	165.3	27	21.6%	61	64	6	3	8	10
2462	Bytom	418	256.0	165.4	101	24.2%	175	243	24	20	17	40
2463	Chorzów	268	250.8	164.5	57	21.3%	107	161	13	5	12	27
2403	cieszyński	1996	1124.3	745.5	146	7.3%	825	1171	18	12	37	79
2464	Częstochowa	888	408.2	241.3	180	20.3%	292	596	36	20	45	79
2404	częstochowski	485	361.7	243.3	83	17.1%	172	313	13	10	23	37
2465	Dąbrowa Górnicza	206	174.2	120.9	40	19.4%	90	116	13	8	8	11
2466	Gliwice	558	315.2	201.7	159	28.5%	199	359	80	18	34	27
2405	gliwicki	410	355.2	256.3	77	18.8%	169	241	33	12	13	19
2467	Jastrzębie-Zdrój	344	390.7	260.6	71	20.6%	135	209	28	12	15	16
2468	Jaworzno	70	77.5	55.6	15	21.4%	34	36	7	3	1	4
2469	Katowice	815	280.5	178.6	158	19.4%	368	447	62	31	20	45
2406	kłobucki	263	311.9	214.5	54	20.5%	107	156	9	13	9	23
2407	lubliniecki	257	337.7	240.4	66	25.7%	106	151	20	2	14	30
2408	mikołowski	269	271.2	204.2	52	19.3%	135	134	7	11	7	27
2470	Mysłowice	142	190.5	134.6	38	26.8%	59	83	2	11	5	20

2409	myszkowski	215	305.0	209.3	49	22.8%	102	113	9	14	10	16
2471	Piekary Śląskie	153	279.7	185.5	29	19.0%	51	102	7	13	3	6
2410	pszczyński	286	255.8	208.2	51	17.8%	124	162	17	7	15	12
2411	raciborski	460	428.3	301.6	88	19.1%	197	263	35	15	21	17
2472	Ruda Śląska	316	231.6	162.6	64	20.3%	134	182	22	6	8	28
2412	rybnicki	658	841.8	685.6	80	12.2%	400	258	24	20	19	17
2473	Rybnik	1508	1099.7	878.2	149	9.9%	943	565	54	39	40	16
2474	Siemianowice Śląskie	83	125.2	80.1	19	22.9%	37	46	9	1	4	5
2475	Sosnowiec	389	196.9	125.6	82	21.1%	159	230	20	22	13	27
2476	Świętochłowice	146	297.3	181.2	25	17.1%	44	102	9	6	1	9
2413	tarnogórski	363	257.8	172.0	74	20.4%	134	229	16	10	21	27
2477	Tychy	355	279.8	191.0	87	24.5%	132	223	21	15	15	36
2415	wodzisławski	932	595.2	423.4	158	17.0%	404	528	49	26	47	36
2478	Zabrze	473	276.7	174.7	84	17.8%	180	293	25	12	18	29
2416	zawierciański	458	392.6	257.5	85	18.5%	199	259	14	10	8	53
2479	Żory	273	434.4	353.2	46	16.8%	153	120	19	10	9	8
2417	żywiecki	408	268.1	185.5	85	20.8%	137	271	12	16	19	38

B. Number of hospitalized due to COVID-19 and dead COVID-19 patients, values of crude and standardized rates of morbidity due to COVID-19 (n/100,000), and values of in-hospital mortality due to COVID-19 in the Silesian Voivodeship in 2021 by poviats

Year		2021										
Code of poviat	Poviat	Number of hospitalized patients	Crude morbidity rate [n/100,000 inhabitants]	Standardized morbidity rate [n/100,000 inhabitants]	Number of dead patients	In-hospital mortality [%]	Number of hospitalized patients age <65 years	Number of hospitalized patients age 65+ years	Number of dead patients without comorbidities	Number of dead patients with one coexisting disease	Number of dead patients with two coexisting disease	Number of dead patients with three coexisting disease
2401	będziński	1145	781.4	515.4	302	26.4%	439	706	74	151	23	54
2402	bielski	1174	705.5	538.9	263	22.4%	462	712	43	52	71	97
2461	Bielsko-Biała	1408	834.0	811.4	279	19.8%	457	951	60	60	73	86
2414	bieruński-lędziński	386	644.4	518.8	96	24.9%	168	218	25	19	18	34
2462	Bytom	1009	621.9	410.5	273	27.1%	341	668	72	73	50	78

2463	Chorzów	875	823.4	580.7	243	27.8%	323	551	62	38	52	91
2403	cieszyński	1748	987.4	704.9	366	20.9%	637	1111	49	64	67	186
2464	Częstochowa	2573	1191.7	763.5	490	19.0%	928	1645	92	62	111	225
2404	częstochowski	1279	957.0	681.4	246	19.2%	467	812	50	31	49	116
2465	Dąbrowa Górnicza	1037	882.4	568.6	257	24.8%	384	653	71	96	28	62
2466	Gliwice	1475	838.0	562.1	388	26.3%	562	913	151	69	97	71
2405	gliwicki	811	704.0	515.1	215	26.5%	335	476	71	38	63	43
2467	Jastrzębie-Zdrój	784	897.8	565.9	256	32.7%	221	563	82	58	56	60
2468	Jaworzno	702	781.5	517.5	229	32.6%	242	460	90	34	49	56
2469	Katowice	2037	704.4	462.9	577	28.3%	799	1238	189	155	49	184
2406	kłobucki	558	664.4	483.0	111	19.9%	224	334	16	66	11	18
2407	lubliniecki	468	617.2	454.9	116	24.8%	193	275	14	15	23	64
2408	mikołowski	621	625.7	484.1	177	28.5%	262	359	43	47	30	57
2470	Mysłowice	504	677.3	513.3	130	25.8%	240	264	30	39	13	48
2409	myszkowski	537	765.9	537.7	137	25.5%	224	313	24	23	33	57
2471	Piekary Śląskie	398	730.7	489.7	100	25.1%	146	252	16	50	10	24
2410	pszczyński	583	521.7	434.6	138	23.7%	259	324	38	22	28	50
2411	raciborski	697	651.6	454.1	206	29.6%	277	420	90	21	47	48
2472	Ruda Śląska	880	648.5	459.0	218	24.8%	335	545	55	45	44	74
2412	rybnicki	643	823.8	639.3	149	23.2%	264	379	52	29	44	24
2473	Rybnik	1177	862.5	630.3	274	23.3%	487	690	93	68	57	56
2474	Siemianowice Śląskie	336	510.3	331.5	98	29.2%	122	214	22	30	17	29
2475	Sosnowiec	1775	905.7	545.3	473	26.6%	599	1176	138	169	94	72
2476	Świętochłowice	362	741.5	514.8	93	25.7%	131	231	23	11	19	40
2413	tarnogórski	992	704.6	504.0	240	24.2%	398	593	43	55	45	97
2477	Tychy	964	764.0	516.3	289	30.0%	359	605	71	52	50	116
2415	wodzisławski	1163	745.7	525.4	334	28.7%	423	740	105	54	87	88
2478	Zabrze	1328	782.0	526.4	308	23.2%	541	787	97	60	78	73
2416	zawierciański	865	746.0	475.5	189	21.8%	295	570	19	31	17	122
2479	Żory	491	781.7	590.2	118	24.0%	189	302	33	30	32	23
2417	żywiecki	1090	718.4	533.9	267	24.5%	434	656	44	51	89	83

International Journal of
Environmental Research and Public Health

MDPI

Supplement 2

Number of hospitalized due to COVID-19, in the Silesian Voivodeship in 2020 and 2021, by municipalities

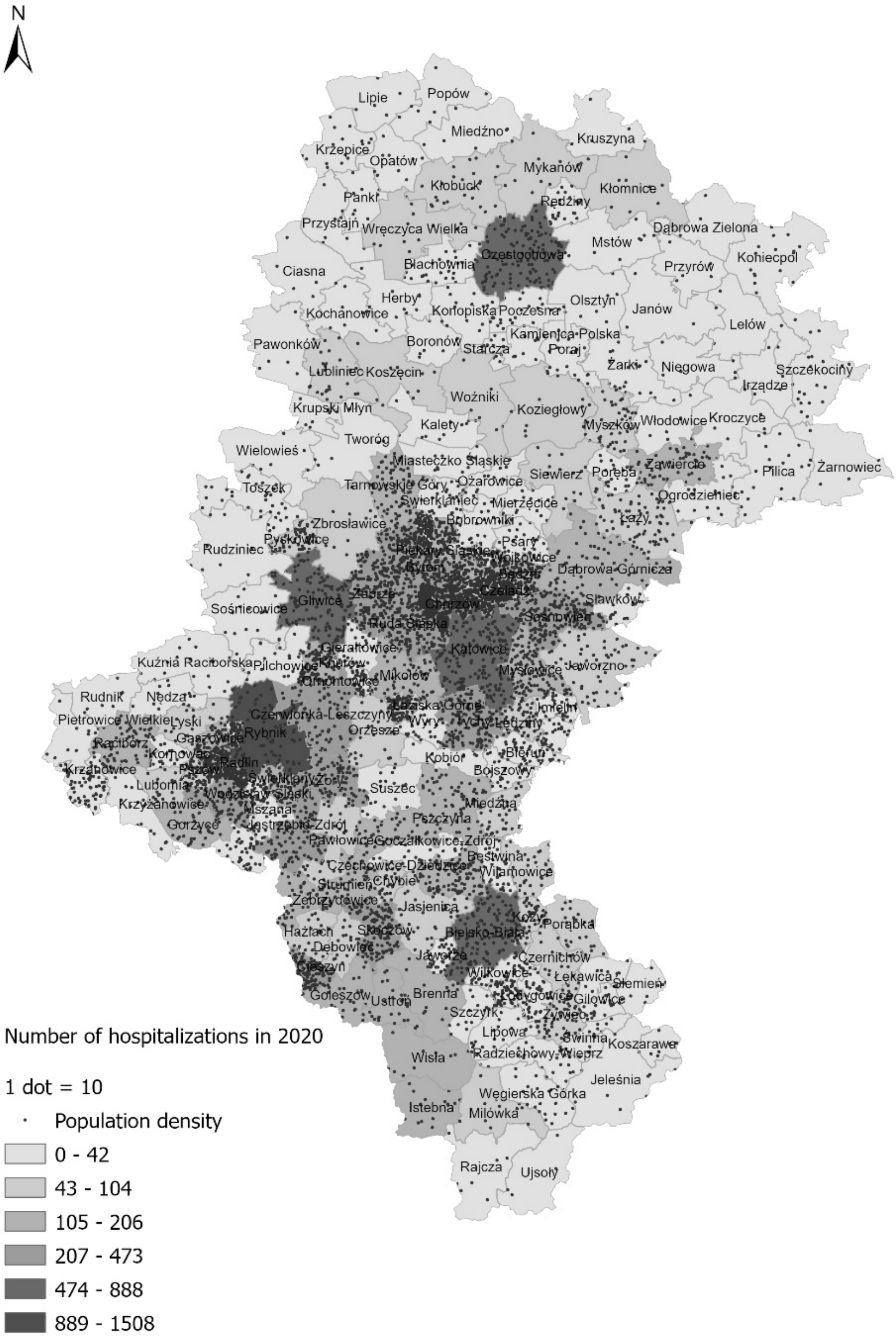

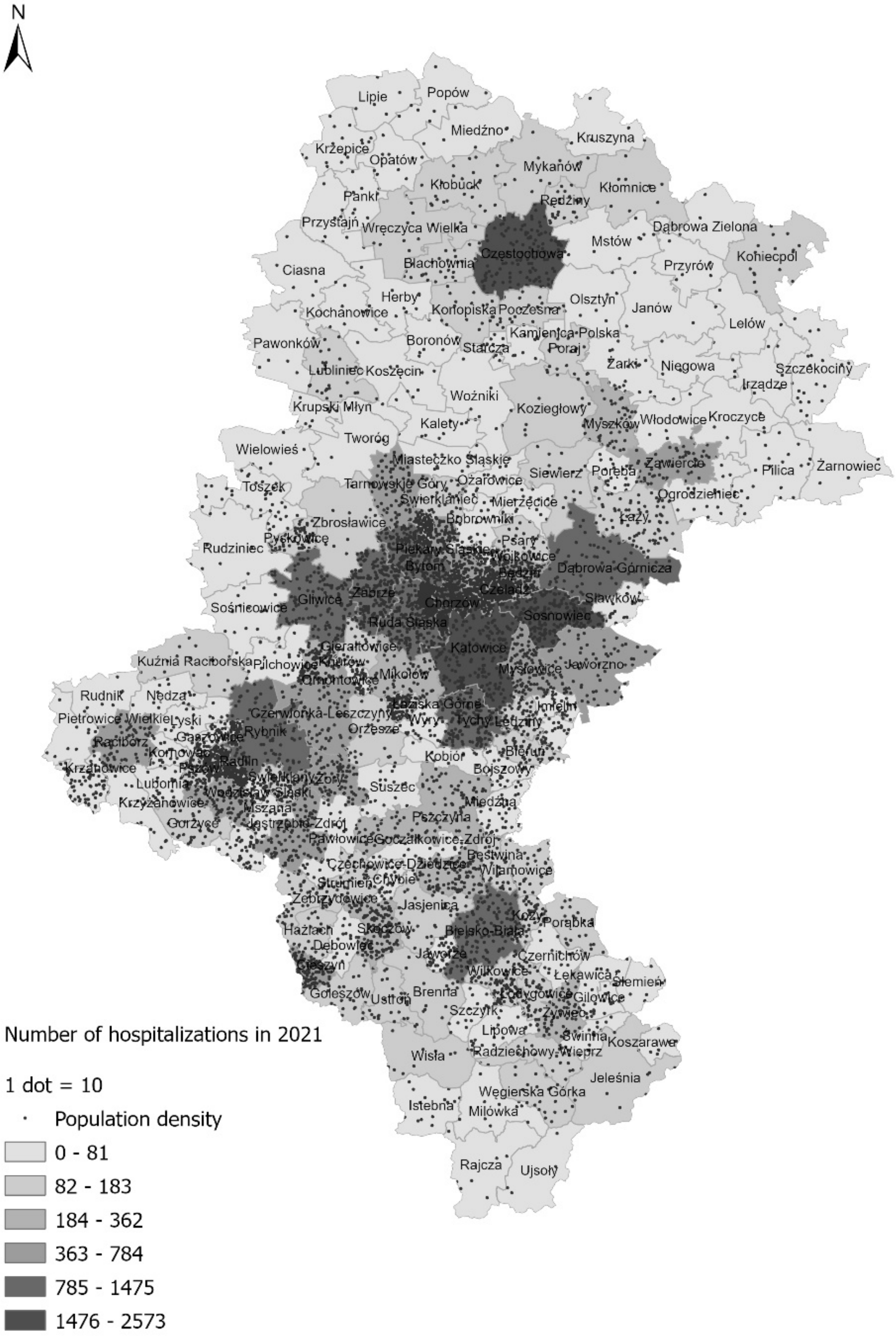
N
Lipie
Popów
Miedźno
Kruszyna
Krzepice
Opatów
Mykanów
Kłobuck
Kłomnice
Panki
Rędziny
Przystajń
Wręczyca Wielka
Dąbrowa Zielona
Częstochowa
Mstów
Blachownia
Koniecpol
Ciasna
Przyrów
Herby
Olsztyn
Kochanowice
Konopiska
Poczesna
Janów
Lelów
Boronów
Kamienica Polska
Pawonków
Starcza
Poraj
Żarki
Niegowa
Szczekociny
Lubliniec
Koszęcin
Irządze
Woźniki
Krupski Młyn
Koziegłowy
Myszków
Włodowice
Kroczyce
Kalety
Tworóg
Wielowieś
Miasteczko Śląskie
Siewierz
Poręba
Zawiercie
Pilica
Żarnowiec
Toszek
Tarnowskie Góry
Ożarowice
Świerklaniec
Mierzęcice
Ogrodzieniec
Łazy
Zbrosławice
Bobrowniki
Pyskowice
Psary
Rudziniec
Piekary Śląskie
Bytom
Wojkowice
Dąbrowa Górnicza
Będzin
Czeladź
Gliwice
Zabrze
Sławków
Sośnicowice
Chorzów
Sosnowiec
Ruda Śląska
Gierałtowice
Katowice
Kuźnia Raciborska
Pilchowice
Knurów
Mysłowice
Jaworzno
Ornontowice
Mikołów
Rudnik
Nędza
Łaziska Górne
Imielin
Pietrowice Wielkie
Lyski
Czerwionka-Leszczyny
Wyry
Tychy
Lędziny
Gaszowice
Orzesze
Racibórz
Rybnik
Kornowac
Bieruń
Kobiór
Radlin
Bojszowy
Krzanowice
Pszów
Świerklany
Żory
Lubomia
Suszec
Wodzisław Śląski
Krzyżanowice
Mszana
Miedźna
Gorzyce
Pszczyna
Jastrzębie-Zdrój
Pawłowice
Goczałkowice-Zdrój
Bestwina
Czechowice-Dziedzice
Wilamowice
Strumień
Chybie
Zebrzydowice
Jasienica
Kozy
Porąbka
Hażlach
Skoczów
Bielsko-Biała
Dębowiec
Jaworze
Czernichów
Cieszyn
Wilkowice
Łękawica
Ślemień
Goleszów
Ustroń
Brenna
Łodygowice
Gilowice
Szczyrk
Żywiec
Lipowa
Świnna
Koszarawa
Wisła
Radziechowy-Wieprz
Jeleśnia
Węgierska Górka
Istebna
Milówka
Rajcza
Ujsoły
Number of hospitalizations in 2021
1 dot = 10
Population density
0 - 81
82 - 183
184 - 362
363 - 784
785 - 1475
1476 - 2573
